# Recent advancements of wastewater treatment by photocatalysis: A comprehensive review

**DOI:** 10.1016/j.isci.2026.116088

**Published:** 2026-05-28

**Authors:** K. Rajanikara Swamy, G. Shyamala, Thirumala Rao Gurugubelli, Gobinath Ravindran, Babu Bathula

**Affiliations:** 1 Department of Civil Engineering, SR University, Warangal, Telangana 506371, India; 2 Department of Basic Sciences, School of Sciences and Humanities, SR University, Warangal, Telangana 506371, India; 3 Symbiosis Center for Research and Innovation (SCRI), Symbiosis International (Deemed University), Pune, Maharashtra, India

**Keywords:** Applied sciences, Engineering, Water resources engineering

## Abstract

The global need for clean water has led to the study of novel treatment methods, and nanotechnology is one of them as it has a high reactivity and a large surface area. Nanomaterials including titanium dioxide (TiO_2_), zinc oxide (ZnO), and carbon nanotubes have shown great efficiency in the removal of heavy metals, dyes, and organic pollutants via photocatalytic reactions as a sustainable water treatment process. This review critically analyzes these nanomaterials, with emphasis on metal oxide-based photocatalysts to degrade recalcitrant contaminants in wastewater. It also discusses synthesis methods, such as top-down and bottom-up methods, their working mechanisms, and applications. Ongoing challenges include large-scale production, cost effectiveness, and optimization of synthesis, which have to be addressed further. The review provides a general overview between the material design, photocatalytic activity, and environmental impact and considers the scalability, industrial viability, and long-term sustainability, thus presenting a balanced evaluation of the studies performed on a large scale.

## Introduction

The situation concerning water shortage and wastewater management is worsening globally, as it is predicted that by 2025, 1.8 billion people will live in water scarcity. According to the World Health Organization (WHO), by 2030, the freshwater demand is anticipated to grow by 30%, and the amount of raw sewage might grow by 80%.^1^ Therefore, more than three-quarters of the people lacking access to well-developed water supply systems are required to manage wastewater efficiently.^2^ The growing need to use clean and safe water due to the high rate of industrialization, urbanization, and population increase has heightened the necessity to come up with effective treatment techniques to eliminate toxic pollutants from wastewater.^3^ Heavy metals, synthetic dyes, and poorly treated industrial effluents are among the main factors that have led to the decline in water quality, and this has caused serious environmental and health risks.^4^^,^^5^ The traditional wastewater treatment methods are known to have a number of drawbacks, such as the lack of elimination of recalcitrant organic contaminants, inadequate elimination of heavy metals and pathogens, production of toxic disinfection by-products, high energy and chemical loads, and sludge management problems, which make them less feasible and dependable in different wastewater environments. Hence, there is a need to opt for tertiary treatment, including ozonation, photocatalytic degradation, and membrane filtration.^6^^,^^7^

The use of nanomaterials in water treatment has attracted much interest because of their properties such as size, surface area, and toxicity related to water handling. Owing to these characteristics, nanomaterials can adsorb a wide range of pollutants, such as heavy metals, dyes, pesticides, and bacteria, in wastewater.^8^^,^^9^ Nanomaterials are utilized in water treatment, mainly wastewater treatment, because they are nano-sized and have larger surface area boundaries; they have larger uptake capacities or adsorption capacities, which aid removal of pollutants including heavy metals, organics, inorganic anions, and bacteria.^10^^,^^11^ Nanomaterial treatment is employed to extend the delivery system of the solution, which is the functional mechanism that maintains the production and supply of reactive oxidizing species in pollutant degradation. Nanomaterials increase and extend the process by offering high surface area, catalytic reactivity, and stability to enable persistent and efficient removal of pollutants.^12^ Among the nanomaterial-based semiconductors used in photocatalysis, such as titanium dioxide (TiO_2_), iron oxide (Fe_2_O_3_), silicon dioxide (SiO_2_), zinc sulfide (ZnS), and zinc oxide (ZnO), the most promising are those based on TiO_2_ and ZnO due to their high activity in the oxidative degradation of organic pollutants and their effectiveness in treatment processes of heavy metals, dyes, pesticides, and bacteria contained in wastewater.^13^^,^^14^^,^^15^ The materials offer the convenience of a 3-fold function as an adsorbent, a catalyst, and an antimicrobial to treat various forms of pollutants in water and to enhance the efficacy of the treatment procedure.^16^ The mechanism of action of nanoparticles is that they produce reactive oxygen species (ROS) and hydroxyl radicals (OH) when they come into contact with UV or visible light; therefore, they photodegrade organic pollutants, thus helping to convert toxic pollutants into substances that are not harmful to the environment, carbon dioxide (CO_2_), and water (H_2_O).^17^^,^^18^

Among the most chemically stable nanoparticles, TiO_2_ is biologically inert and more oxidative in aqueous media and applied in water purification and industrial wastewater treatment.^10^ Moreover, TiO_2_ nanoparticles have been incorporated into different types of composite materials to increase their photocatalytic activity and stability.^19^ As an example, TiO_2_/PVDF (polyvinylidene fluoride) composite membrane is used in the high degradation efficiency of brilliant green and indigo, carmine dyes, and TiO_2_ (polyacrylonitrile) composite used in the inefficient decolorization of Rhodamine-B under UV light.^20^^,^^21^ Besides TiO_2_, other semiconductors and metal oxides also invoke the principle of nanocatalysts, expanding the properties of large surface/volume ratios and greater catalytic reactivity.^22^^,^^23^ Such nanocatalysts are applied for the decontamination of a broad category of pollutants (metal ions and organic compounds) through advanced oxidation processes.^24^^,^^25^ Nanotubes, nanofibers, and nanomembranes are also used to remove heavy metal ions and other pollutants; they make a very efficient method of water.^16^ Moreover, copper and silver nano-metal oxides have been utilized because of their antimicrobial activity and ability to photocatalytically breakdown organic pollutants.^8^ There are some disadvantages to using free nanoparticles in suspension format, which are aggregation issues and separation problems that diminish their efficiency and add to the hazardous effect on the environment and human health.^26^ To address these shortcomings, different composites have been suggested, including polymer-encapsulated nanoparticles and alginate beads, in which agglomeration of nanoparticles is reduced and nanoparticles are obtained without difficulty and can be recycled.^27^ As an example, nanoparticles, such as nanoscale zero-valent iron (nZVI) and Fe_3_O_4_, have been immobilized on alginate beads because they enhance their stability and are easily separable after use in treated water.^28^ Another natural polymer, chitosan, is employed to prepare composite beads with nanoparticles to adsorb metal ions and organic contaminants. The latest developments in photocatalytic wastewater treatment relate to the development of effective photocatalysts, including biocatalysts, C_3_N_4_ (carbon nitride), and ZnO with efficient band gap properties, which facilitate enhanced degradation of organic contaminants.^29^

**Figure 1:**
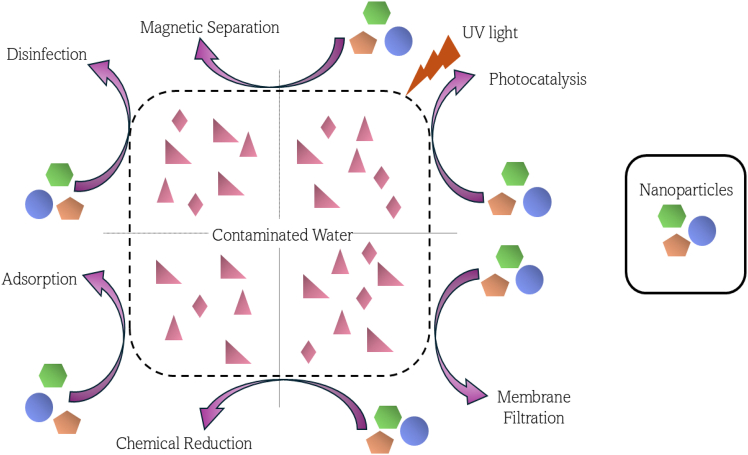
Various applications of nanoparticles in wastewater treatment Licensed under CC BY 4.0.^12^

Nanoparticle use in wastewater treatment (Figure 1) is associated not only with an increase in the efficacy of pollutant removal but also with the elimination of the drawbacks of traditional water treatment methods, such as reverse osmosis and chlorination, which increase costs and produce undesirable by-products.^8^ Furthermore, the use of photocatalysis in combination with other treatment processes, including adsorption and membrane technologies, is a potential strategy for improving the performance of wastewater treatment plants (WWTPs).^16^^,^^30^ However, there are still some concerns regarding the commercialization and cost effectiveness of the application of nanomaterials in water treatment. Further work is required to enhance the properties of nanomaterials to remove pollutants, guarantee their biophysical compatibility, and reduce the cost associated with the production of nanomaterials to address the global challenges of water scarcity and pollution.

The present review is a multi-dimensional framework that synthetically couples the synthesis routes (physical, chemical, and biological), structural and morphological engineering, defect chemistry and band gap tuning, photocatalytic processes, and reactors. This nexus of structure, property and performance, and sustainability has barely received literature discussion as a concept. A critical comparative analysis of TiO_2_, ZnO, and hybrid systems of pure materials, doped materials, heterojunction structures, and green-synthesized nanomaterials is also given in the review, with direct comparisons of the performance of degradation between different materials under different operating conditions. Moreover, it highlights green synthesis and ecotoxicology by combining bio-assisted synthesis technology; statistical optimization technology, including long-term environmental hazards; and nanoparticle destiny in WWTPs—issues that have been considered separately in many studies. Notably, the review not only evaluates the laboratory results regarding the scalability and industrial translation by analyzing the strategies applied in the recovery (e.g., magnetic supports and polymer immobilization) but also covers the combinations of photocatalysis and membranes or adsorption, cost-effectiveness limitations, and constraints at the reactor level. Lastly, it summarizes the latest developments during 2023–2025 such as heterojunction engineering, visible-light activation, composite and defect-rich nanostructures, and sustainable nanomaterial synthesis, which places the work as a prospective and application-focused synthesis and not a descriptive overview.

## Synthesis of nanoparticles

**Figure 2:**
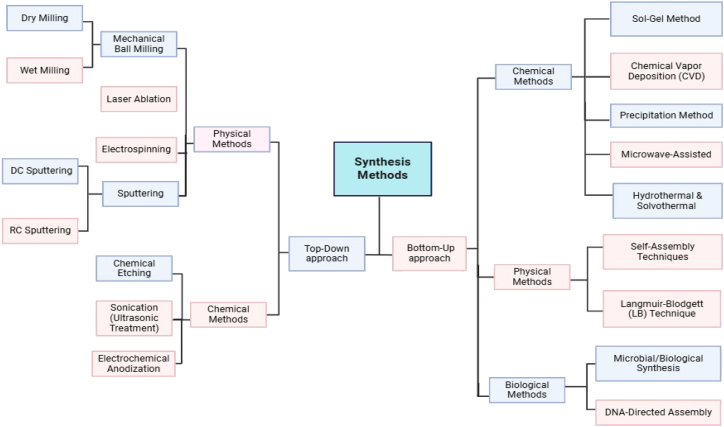
Classification of top-down and bottom-up synthesis approaches for nanoparticles Adapted with permission from Mondal et al.^25^ Copyright 2023 Elsevier.

The synthesis of nanoparticles determines their properties, such as size, shape, stability, and functionality. There are two basic techniques for the preparation of nanoparticles, top-down and bottom-up approaches, as shown schematically in Figure 2, and they influence the properties in different ways.^31^ The top-down approach involves the conversion of large volumes of materials into nanoparticles, which often results in morphological distortions owing to mechanical means.^32^ However, the bottom-up approach, which involves chemical and biological methods, provides a lot of control over the size and morphology of nanoparticles, as particles are made using atoms or molecules.^33^ This method is useful for producing nanostructures with uniform physicochemical characteristics.^34^ For example, in the sol-gel technique, a bottom-up technique, the size of nanoparticles and their distribution are managed; however, the chemicals used are toxic to the environment and pose health hazards to humans.^32^

### Physical synthesis

Physical synthesis methods of nanomaterials are physical vapor deposition, laser ablation, sputtering, and ball milling.^35^ These techniques typically involve high-energy sources; therefore, they employ heterogeneous nanoparticles. Recent literature has indicated the obvious trend of moving away from the classical, energy-intensive physical routes (flame/aerosol, high-temperature thermal decomposition, and UV/heat breakdown) in favor of solution-based procedures along with hybrid physical-assisted procedures that provide finer control of morphology at reduced cost and environmental impact.^36^^,^^37^^,^^38^ Conventional physical methods are capable of synthesizing high-purity TiO_2_ and ZnO but require extremely high temperatures, pressures, and complicated equipment and cannot be made sustainably. These are one of the primary reasons to utilize green and wet chemical methods more often, in particular when working with doped and composite systems when a fine regulation of interfaces is necessary.^37^^,^^39^

Recent advances in physical and semi-physical preparation procedures of TiO_2_-ZnO nanocomposites are focused on morphology, defect chemistry, and interfacial structure control with accuracy. Liquid laser ablation allows the synthesis of TiO_2_-ZnO nanostructures of high purity and ligand free through the ablation of Ti and Zn, using pulsed lasers in water. Direct control of the electronic structure, such as Ti^3+^/Ti^2+^ self-dope levels, is realized by pulse power and ablation sequence and correlates physical parameters with visible-light photocatalytic activity.^40^ But still, liquid laser ablation is limited by the low throughput and high capital cost, which do not allow scaling the industry. Ultrasonic energy is applied to produce a product by Sono synthesis and sonication-assisted methods, either as the main driver of synthesis or as a dispersion/doping agent. Such process characteristics are the generation of 20–30 nm TiO_2_, ZnO, or Fe_3_O_4_/TiO_2_ composites with enhanced antibacterial and photocatalytic activity by utilizing low-temperature (100°C), rapid (3–4 h), and equipment-simplified processes.^41^^,^^42^ However, the reproducibility and particle size distribution are highly related to the sonication power and reactor geometry. Thermal treatment and electrospinning of TiO_2_/ZnO form heterojunction fibers with engineered architectures, and smaller band gaps (approximately 1.17 eV) were used to enhance charge separation and recyclability.^43^^,^^44^ However, multistep processing and energy-consuming calcification continue to be the major disadvantages. Low-cost scalability Simple mechanical mixing and milling methods have low interfacial control but are simple to scale to high performance, typically necessitating additional surface modification to reach solution-based route performance.^45^^,^^46^^,^^47^

In comparison to classical physical routes, solution-based (sol-gel, hydrothermal, and precipitation) and green biosynthetic cap routes prevail because they have a high level of morphology tunability, band-gap engineering, and moderate processing conditions.^37^^,^^38^ The concept of green synthesis also aims at energy intensity and the risk of dangerous by-products of traditional physical methods.^36^^,^^39^ Hybrid strategies that include the integration of the structural precision of physical methods with the scalability and sustainability of green synthesis routes will result in the desired advancements in the future. The synthesis pathway of nanoparticles is a determining factor for regulating their morphology, crystallinity, surface charge, and eventually their photocatalytic efficiency. Between the two general synthesis strategies used to synthesize them, namely the top-down and the bottom-up, the latter is typically more favorable to photocatalytic applications, as it provides a much greater control of the particle size and surface functionalization. Nevertheless, top-down processes like ball milling or sputtering, though scalable and simple, tend to cause surface defects and highly uneven size distribution and are energy intensive to process, which restrict its application to large-scale wastewater treatment.

### Chemical synthesis

**Figure 3:**
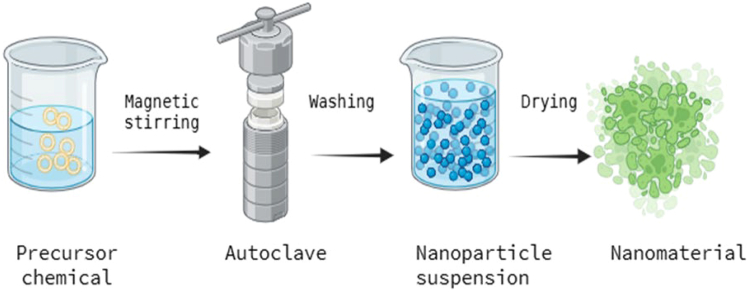
Process of hydrothermal synthesis of nanomaterials

The various chemical synthesis routes to synthesize metal oxide nanoparticles are sol-gel, hydrothermal, precipitation, template-assisted, spray pyrolysis, chemical vapor deposition (CVD), etc. They are distinguished by great flexibility and rather low cost; the size, shape, and crystalline structure of the nanoparticles prepared by this method are easily adjusted by simply changing the reaction conditions, such as temperature, pressure, and pH of the solution.^14^^,^^48^ Hydrothermal synthesis, illustrated in Figure 3, is a simple and safe technique that does not involve harmful reagents and is applied in the preparation of many nanoparticles.^6^^,^^49^

Sol-gel and solution-based methods are still considered the underlying ones, as they provide high purity, tunable particle size, porosity, and control over the phase at comparatively low temperatures.^37^^,^^50^ As an example, ZnO-doped TiO_2_ is formed using sol-gel with a specific morphology and increased visible light-driven photocatalytic and antimicrobial properties.^51^ Sol-gel techniques with ultrasound further enhance precursor mixing and the uniformity of gels, making it possible to have hierarchical TiO_2_-ZnO/chitosan-graphene systems to degrade antibiotics.^52^ Notably, low-temperature crystalline TiO_2_ with low organic content can now be obtained in aqueous colloidal sol-gel without high-temperature calcination and is performing similarly to a commercial P25.^53^ The hydrothermal and solvothermal techniques have become the main focus in the morphology-engineered nanostructures. Relatively mild and, moreover, water-based conditions, allow nanowires, nanorods, and mesoporous TiO_2_-ZnO heterostructures to be prepared using these routes.^37^^,^^54^ Interface-engineered systems, including epitaxially grown ZnO nanowires on TiO_2_, show that controlled nucleation and growth chemistry enhance the crystallinity, quality of the heterojunction, and multiple functionalities, including photocatalysis and dye sensitization of solar cells.^55^ Accurate regulation of precursor chemistry, pH, mineralizers, and growth-guiding agents, and not the formation of particles, is what establishes the critical advancement.^37^ Precipitation and co-precipitation have been repackaged and made into controllable and green-friendly pathways. One of the biggest changes is the emergence of bio-assisted and green synthesis. Conventional chemical approaches usually rely on poisonous solvents and stabilizers, which is why the shift of focus to plant extracts, biogenic surfactants, and other biological agents as reducing, capping, and structure-directing agents has been made.^36^^,^^39^^,^^56^^,^^57^

Zn-doped TiO_2_ with increased visible light absorption and antimicrobial characteristics has been obtained by both green hydrothermal and sol-gel approaches.^36^ It has been comparatively demonstrated that TiO_2_ synthesized in green through orange peel extract is equivalent to or even better than traditional sol-gel TiO_2_ in terms of crystallite size control and tuning of band gap.^58^ However, biological precursor variability, incomplete mechanistic knowledge, and scale-up issues are also the critical gaps in research.^39^^,^^59^ Electronic structure engineering is also becoming subject to chemical synthesis. By using chemical reduction to create black TiO_2_, mid-gap states are generated to facilitate visible light activity.^37^ Sol-gel Nb^5+^-doped TiO_2_ is observed to be tuned in band structure and stabilized in anatase, which is enabled by density functional theory (DFT) analysis.^60^ Co-doping with green chemistry (e.g., Zn/Mg in TiO_2_) is done with the help of biogenic surfactants.^57^ Likewise, the progress in ZnO production through sol-gel, precipitation, and microemulsion techniques has enabled accurate morphology and surface regulation, but the environmental issues still lead to hybridization with more environmentally friendly procedures.^61^^,^^62^

In general, the process of chemical synthesis has become eco-aware and multi-purpose in its design. It has advantages such as outstanding phase, morphology, doping, and interface control and hybrid-strategy compatibility.^50^^,^^55^ Yet, the problems of solvent toxicity, variability of the batch in green routes, and incomplete life-cycle assessments (LCAs) like those are still present.^36^^,^^39^^,^^59^ The integration of the principles of green chemistry, statistical optimization, and defect engineering into scale-based manufacturing models will become the future.

### Biological synthesis

**Figure 4:**
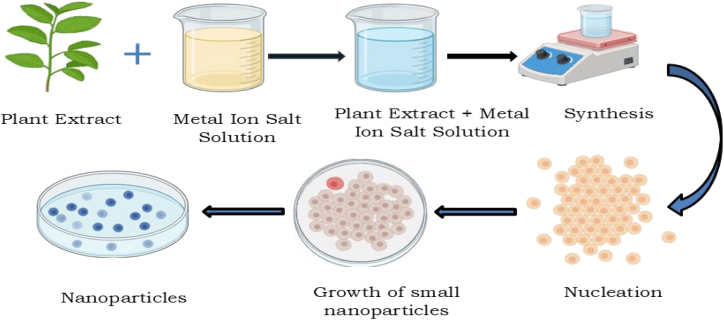
Mechanism of production of nanoparticles from plant extracts

Biological synthesis includes the use of plant parts such as leaves, seed bark, and plant extracts to synthesize metal oxide nanoparticles. Reducing, stabilizing, and capping agents used in solution-based synthesis methods (such as solgel, hydro/solvothermal, and co-precipitation techniques) are biological reductants, which include plant extracts, microorganisms, and biomolecules like proteins, enzymes, and phytochemicals.^39^^,^^63^^,^^64^^,^^65^^,^^66^ Metal ion reduction, nucleation, growth, and stabilization are controlled by phytochemicals and proteins, which effectively biomimic biological systems to be considered as bionanofactories. This method is in contrast to the traditional solvent-intensive and reductant-intensive chemical routes with toxic byproducts and energy-intensive mechanisms.^67^^,^^68^ Green synthesis is a method of nanoparticle production in which bacteria, fungi, algae, or plant extracts are used to reduce and stabilize agents, as depicted in Figure 4.^33^^,^^69^

Green nanomaterials that shift toward performance rather than eco-labels are one of the notable transformations. Bio-derived surface properties and defect structures are said to provide biologically synthesized TiO_2_ and ZnO with similar or even better photocatalytic, antibacterial, and antioxidant properties than chemically prepared counterparts.^65^^,^^66^ Plant-based TiO_2_ is an example that showed better dye degradation and antibacterial performance despite phase composition and crystallite size similar to those of chemically prepared TiO_2_.^65^^,^^67^ Equally, green ZnO nanoparticles have good antimicrobial, catalytic, and UV-filtering properties without the use of synthetic surfactants and capping agents.^68^^,^^70^^,^^71^

Synthesis of TiO_2_ is currently addressed in photosynthesis with wastes of plants and agricultural applications as well as in microbial pathways with bacteria, fungi, algae, yeast, and even viruses.^66^^,^^72^ Such pathways make use of bioactive metabolites to guide the formation and stabilization of nanoparticles. In the case of ZnO, green synthesis has advanced to biomolecule-specific templating (e.g., ovalbumin-assisted synthesis of well-crystallized 8–14 nm ZnO with improved antimicrobial activity proves protein-templated nanocrystal engineering).^68^^,^^73^

The benefits behind adoption have been the expansion of environmental and safety profiles and water-based processing, as well as natural reducing agents, biocompatible, cleaner particle surfaces, which have biomedical applications, and functionally rich bio-capping layers to dispersion, stability, and ROS.^59^^,^^66^ Also, the abundance of waste plant use and the use of low-tech equipment indicate possibilities of realistic scaling.^74^ Despite reproducibility, a lack of mechanistic understanding of biomolecule-metal interactions, scale-up issues, as well as an incomplete assessment of long-term toxicity and life cycle are important obstacles.^63^^,^^75^ The different techniques used for synthesizing nanoparticles are presented in Table 1. The future direction will be based on making omics and AI-based tools correlate the extract composition with the nanoparticle characteristics, creating standard protocols, and carrying out systematic comparative toxicity and sustainability studies in comparison with chemical and physical synthesis pathways.

**Table 1:** Synthesis of various types of nanoparticles using different methods

Morphology	Synthesis method	Synthesis condition		Description	Reference
		Temperature (°C)	pH condition		
Nanorods	co-precipitation, sol-gel, and adsorption-based nanocomposites	25°C–200°C	8–11 (alkaline)	allows for the controlled growth of nanorods with specific dimensions 10–130 nm; the resulting nanorods exhibit improved structural stability and high surface area.	Ghosh et al.^4^
Nanotubes	chemical precipitation, sol-gel, and green synthesis	80°C–200°C	6–12 (material dependent)	producing high-purity CNTs, as it minimizes contamination during the synthesis process.	Epelle et al.^76^
Nanosheets	self-assembled method	room temperature to 80°C	4–10	thin, flat structures with large surface areas as much as 265.456 m^2^/g.	Tou et al.^12^
Nanoring	hydrothermal (commercially synthesized)	100°C	7.5 (neutral)	circular structures with a hollow center (diameter of 155 nm, and ring thickness of 40 nm).	Zhang et al.^77^
Nanowires	sol-gel, co-precipitation, hydrothermal, and biological	80°C–200°C	8–12 (alkaline)	long, thin structures with high aspect ratios; their lengths ranged from 2 to 5 μm, with diameters of 20–40 nm.	Alhalili^8^
Nanostars	room temperature precipitation + UV-A photoreduction	25°C (synthesis), 150°C (drying)	12.0 (strongly alkaline)	star-shaped structures with a core and multiple branches; showed broad absorbance patterns in the ranges of 660–775 nm and 930–1000 nm.	Andrade et al.^78^
Nanospheres	mechanical ball milling	typically room temperature (depending on the milling duration)	neutral to slightly alkaline (pH 7–9)	effective for the removal of harmful pathogens and bacteria from water, making it a cost-effective option for wastewater treatment.	Palani et al.^79^
Nanodendrites	synergistic effect of self-assembled functional surfactants and halide ions	room temperature to 80°C	4–9	tree-like branched structures; nanodendrites showed enhanced electrocatalytic activity and stability	Tou et al.^12^
Nanodisks	low-temperature hydrothermal synthesis	90°C–95°C	10–11 (alkaline)	flat, circular structures; ZnO nanodisks showed improved photocatalytic performance due to specific crystal planes	Zeng et al.^80^
Nanopyramids	hydrothermal selenylation of CdO inside porous TiO_2_	180°C	7–8 (neutral to slightly alkaline)	pyramid-shaped structures; CdIn_2_S_4_ nanopyramids exhibited improved photocatalytic properties compared with CdS	Yang et al.^81^

## Physicochemical characterization of TiO_2_ and ZnO nanomaterials: Critical insights from advanced techniques

**Figure 5:**
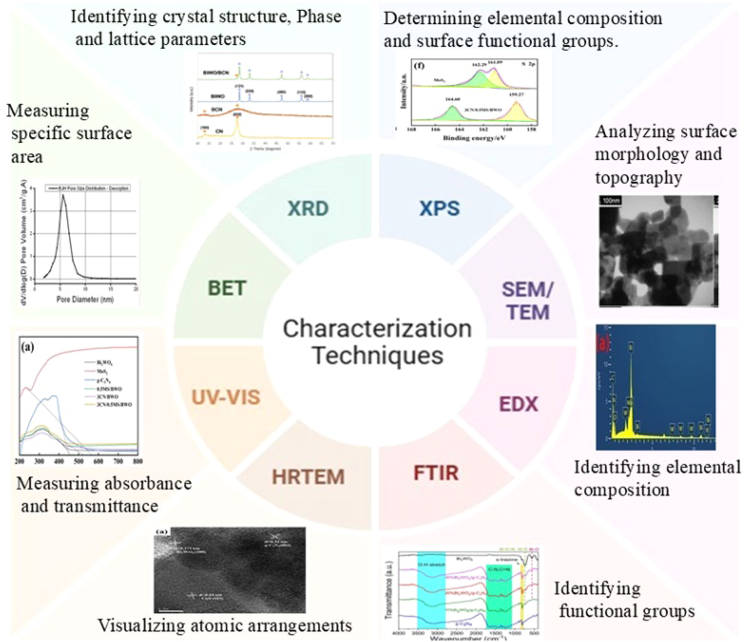
Different characterization techniques used to analyze nanocomposites

The characterization methods used for nanoparticles in the wastewater treatment process are shown in Figure 5. Transmission electron microscopy (TEM) is advantageous for revealing the shape and size of nanoparticles. The crystal structure of the nanoparticles is determined using X-ray diffraction (XRD).^19^^,^^82^ Fourier transform infrared spectroscopy (FTIR) provides information about the functional groups available on the surface of the produced nanoparticles, as well as all functional groups.^83^

Recent studies of TiO_2_ and ZnO nanomaterials indicate that minor modifications of the size, phase, morphology, surface chemistry, and synthesis pathway, which are unveiled by sophisticated characterization, determine their functional responses in catalysis, sensing, coatings, and biomedicine. TiO_2_ and ZnO exhibit a very high degree of correlation between performance and a composite of other characteristics, such as crystal phase, particle size, shape, surface area, porosity, band gap, surface defects, and heterojunction formation. New protocols to characterize these properties are based on the combination of XRD, electron microscopy (SEM/TEM), high-resolution transmission electron microscopy (HRTEM), FTIR, ultraviolet-visible (UV-vis) spectroscopy, Brunauer-Emmett-Teller (BET), X-ray photoelectron spectroscopy (XPS), and electrochemical techniques.^84^^,^^85^^,^^86^ Detailed reviews have now highlighted that individual methods are hardly adequate, and reliable structure-property correlations must be multi-technique fingerprinting of nanoparticles.^84^

### Advanced insights for TiO_2_: Phase, morphology, and defect chemistry

The synthesis technique of inverse microemulsion allows fine control of TiO_2_ polymorphs (pure anatase, pure rutile, or mixtures of anatase/rutile/brookite) easily by changing precursor chemistry, and phase and crystallinity are determined by XRD, Raman spectroscopy, and TEM.^86^ Optical absorption, band gap, carrier concentration, and conductivity with granular and nanofiber TiO_2_ of similar anatase structure exhibit significant differences, with nanofibers having higher aspect ratios and surface areas and better Raman modes, and narrowed band gaps and greater conductivity directly enhancing optoelectronic suitability.^87^ By pH-tuning rotational growth of TiO_2_ nanorods and through XRD, FESEM, and UV-vis spectroscopy, it was demonstrated that an increase in pH results in finer rods and a narrower band gap that correlate with enhanced photocatalytic methyl orange degradation.^88^ Another emerging interest is defect and dopant chemistry that is studied using XPS, FTIR, and photoluminescence. Lattice constants, oxygen vacancy concentration and optical transitions, TiO_2_ reflectance, band gap, and thermal/electrical characteristics are altered by metal doping (Ce, Ag, La, and Cu). However, a significant portion of this is still phenomenological; quantitative correlations between dopant defect populations by XPS/EPR and the dynamics of charge carriers are yet to be developed.^89^^,^^90^

### Advanced insights for ZnO: Green synthesis, doping, and electroactive behavior

In the case of ZnO, defining the way in which structure and surface chemistry are modified by synthetic strategies involving one or both of the methods of being green or complexation is one of the primary themes. ZnO synthesis with the assistance of Moringa (interrogated by XRD, HRTEM/SAED, DSC/TGA, FTIR, UV-vis spectroscopy, and cyclic voltammetry) is highly crystalline, phase-pure ZnO with high electrochemical activity and a multi-step thermal evolution process between organics and ZnO and subsequently into metallic Zn.^91^ This multidimensional description elucidates a formation mechanism linked to certain bioactive ligands and is no longer simply an empirical route that uses the name “green.” Similarly, tea-assisted combustion ZnO was characterized by XRD, FTIR, TEM, UV-vis spectroscopy, and zeta potential, which showed that gentle heat treatment enhances the size of the crystallites, causes narrowing of the band gap to moderate, and allows maintenance of the good colloidal stability; gas sensing tests then relate these physicochemical changes to the enhanced ethanol activity.^92^ The studies are relevant to the way that the integration of spectroscopy/microscopy with application testing leads to mechanistic design principles (e.g., optimum crystallite size and surface chemistry of gas sensors). XRD, FTIR, Raman spectroscopy, UV-vis spectroscopy, and zeta potential are used to study transition metal doping, i.e., Cr-doped ZnO, which shows lattice distortion, size reduction of crystallite, and widening of band gaps but does not lose colloidal stability.^93^ But, like TiO_2_, many studies have not gone beyond correlating an increase or decrease in the band gaps with the doping level, and systematic, defect-free electronic transport and *in situ* operando studies are limited.

### TiO_2_-ZnO hybrids and heterojunction engineering

The example of hybrid TiO_2_-ZnO nanomaterials points to the point at which new, more advanced characterizations indeed transform knowledge. The combination of laser ablation in liquid and TEM, XPS, and UV-vis spectroscopy demonstrates the presence of potentially diverse architectures based on different types of TiO_2_ nanospheres adorning ZnO nanorods and flower-like ZnO aggregates, with a non-monochromatic tunable self-doping of Ti^3+^/Ti^2+^ under laser pulse energy. XPS indicates that the surface chemistry and the concentration of defects (oxygen vacancies and reduced Ti states) are vulnerable to ablation sequence and energy and, thus, can be deliberately engineered to form heterojunctions and defect-rich surfaces, which would be otherwise expected to enhance photocatalytic and gas-sensing behaviors.^40^

The ZnO-TiO_2_ materials that have been prepared chemically also demonstrate the effect of composition and path on determining physicochemical properties. Indicatively, crystallinity, agglomeration, band gap, and morphology are all different when the ZnO/ZnO+TiO_2_ ratio is varied and sol-gel and precipitation are used, evaluated by conventional structural and optical indicators. These parameters also result in non-monotonic variations in photocatalytic degradation of methyl orange, whereby various optimal fractions of ZnO vary depending on the synthesis pathway, highlighting the fact that the same composition does not necessarily translate to the same material without their in-depth characterization.^94^ Combined XRD, Raman, UV-vis, and impedance spectrophotometry reveals how hybridization is associated with ultrasonic TiO_2_@ZnO nanocomposites with rutile TiO_2_ signals in Raman, reduced particle size, increased band gaps, and significant changes in dielectric and AC conductivity.^95^

**Figure 6:**
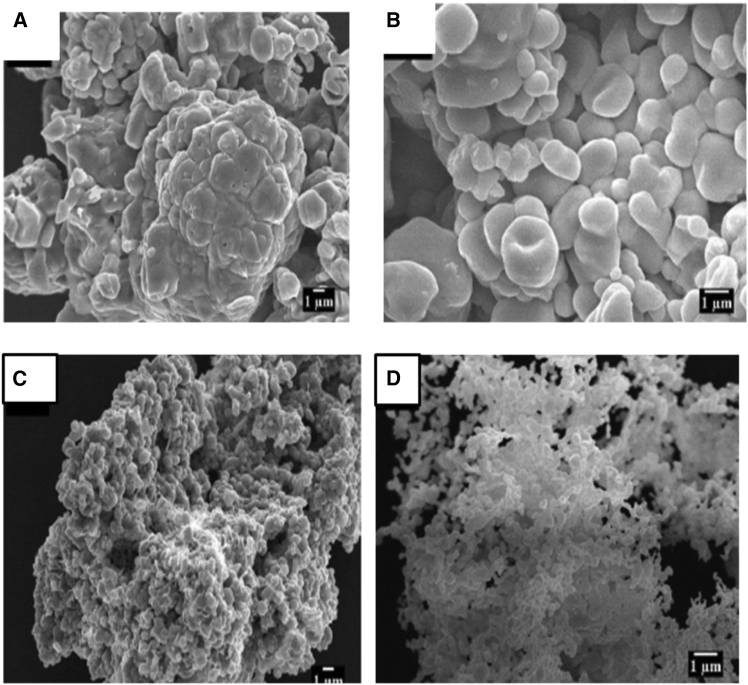
SEM images (A and B) 4,000× (A) and 8,000× (B) images of ZnO nanoparticles obtained using *Aloe Vera* (Zn-AL). (C and D) 4000× (C) and 8,000× (D) images of ZnO nanoparticles obtained from cassava starch (Zn-ST). Licensed under CC BY 4.0.^4^

Elemental analysis of the nanoparticles was conducted using energy-dispersive X-ray spectroscopy (EDX). The saturation magnetization of a particular nanoparticle was determined using a vibrating sample magnetometer (VSM). The thermal stability of a nanoparticle is determined by thermo-gravimetric analysis (TGA).^96^^,^^97^ SEM revealed the outer shape of the nanoparticles, whereas TEM revealed the inside shape of the nanoparticles.^69^ In the present investigation, the functional groups and molecular structures of the synthesized nanocomposites were detected using FTIR spectroscopy, as shown in Figure 6, and the morphologies and surface topographies of the samples were analyzed using SEM.^98^ ZnO nanoparticles were characterized to determine the particle size, and the average crystallite size of the particles was determined using the Debye Scherrer formula from the XRD pattern.^83^^,^^99^

**Figure 7:**
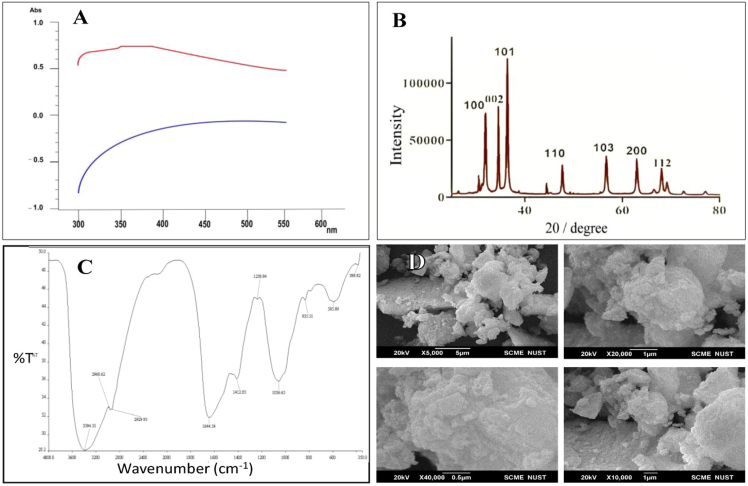
Characterization of the morphology of ZnO nanoparticles (A) UV-vis spectroscopy. (B) XRD. (C) FTIR. (D) SEM. Licensed under CC BY 4.0.^83^

As shown in Figure 7, UV-vis spectroscopy was employed for the optical assessment and nanoparticle identification of absorption bands in studies on ZnO nanoparticles.^9^^,^^83^^,^^100^ Furthermore, nitrogen gas adsorption/desorption measurements were also performed to confirm the BET and DFT for surface areas, pore size, and pore volume.^101^ These techniques are essential for understanding the morphology, magnetic nature, size, shape, and composition of nanoparticles.^102^^,^^103^

**Figure 8:**
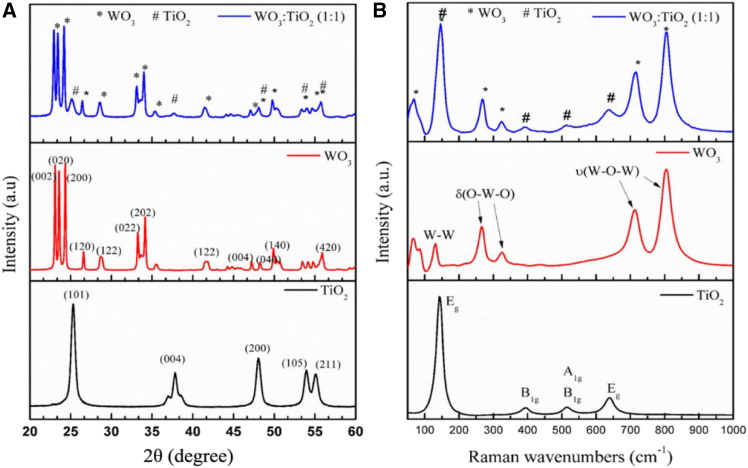
XRD patterns and Raman spectra of TiO_2_, WO_3_, and WO_3_:TiO_2_ (A and B) XRD patterns (A) and Raman bands (B) of TiO_2_, WO_3_, and WO_3_:TiO_2_ (1:1). δ(O-W-O), bending vibration mode; υ(W-O-W), stretching vibration mode. Reproduced with permission from Simelane and Dlamini.^104^ Copyright 2019 Elsevier.

The XRD patterns of the nanoparticles revealed that the tungsten trioxide (WO_3_) phase was monoclinic, while the TiO_2_ phase was anatase, as depicted in Figure 8A. The Raman data shown in Figure 8B support the XRD results.^104^^,^^105^

## Photocatalytic mechanism

Photocatalysis is the process in which a catalyst is “switched on” by light, and it is a metallic oxide semiconductor that reduces or oxidizes species to make them less hazardous.^30^^,^^49^^,^^106^ This is especially true in the breakdown of non-biodegradable hazardous substances, meaning that the improvement of this method is functional for environmental conservation.^26^^,^^34^ Photocatalytic activity is connected with the efficiency of photodegradation.^107^ Photocatalyst-induced processes are promising for water treatment and inactivation of organic materials. TiO_2_ and ZnO have emerged as photocatalysts that have attracted more attention in the recent past due to their chemical non-reactivity, non-toxicity, and potential for generating ROS, which are essential in the degradation of organic pollutants when exposed to light photocatalysis.^29^^,^^79^^,^^108^ This has been explained with examples of different pollutants such as heavy metals and dyes removed from wastewater.^77^^,^^109^ For example, green-synthesized TiO_2_ nanoparticles have the potential to remove chromium and reduce the chemical oxygen demand (COD) in tannery wastewater, which sheds light on the photocatalytic degradation of industrial effluents.^110^ Furthermore, the photocatalytic performance of solar energy in promoting environmental cleaning is sustainable and economically viable, thereby encouraging the broad-scale application of photocatalytic procedures. The synthesis of photocatalysts using green methods is not only environmentally friendly but also increases the photocatalytic activity of the process, as identified by the biosynthesis of ZnO nanoparticles.^109^^,^^111^ Such developments demonstrate that photocatalytic processes could provide solutions to a range of problems in the context of pollution control and the efficient removal of a wide spectrum of recalcitrant compounds from industrial effluents, thus improving the quality of the environment and well being of people.^26^^,^^34^

Photocatalysis is a technology that uses light and other chemicals, such as TiO_2_ and ZnO, to remove pollutants from wastewater.^112^^,^^113^ These activities are postulated to be due to the excitation of electrons and holes (e/h^+^ pairs) or generation of hydroxyl radicals (OH). For example, ZnO-PVP nanoparticles have been shown to have improved photocatalytic performance because of their larger surface area and better relative charge separation, which are vital for the decomposition of pollutants.^114^^,^^115^^,^^116^ These electron-hole pairs are helpful because they cause other redox reactions in the mentioned materials.^109^ Electrons in the conduction band easily neutralize pollutants or even generate more serious oxidants after reacting with oxygen; holes in the valence band oxidize pollutants or react with water to generate hydroxyl radicals.^117^ The generation of hydroxyl radicals is of great importance, as such species are highly reactive, and the reaction of such hydroxyl radicals causes the degradation of multi-component organic molecules into less hazardous forms.^118^ Other factors that influence the overall efficiency of the photocatalytic process include the type of reactor used, light intensity, and contact time.^107^ Additionally, photocatalytic decay processes are influenced by the pH of the solution because they alter the charge distribution on the surface of the photocatalyst and the concentration of reactive species.^119^ Despite the key advantages of chemical-free degradation and compatibility with other treatments, photocatalysis faces the following challenges: catalyst stability, cost, and applicability on the industrial scale. In addition, carbonaceous nanomaterials including CNTs and graphene incorporated with photocatalysts, such as TiO_2_, improve the photocatalytic efficiency resulting from better electron transfer and reduced electron-hole recombination.^38^^,^^120^^,^^121^ The specific mechanism of photocatalysis depends on the species within the solution and the species at the catalyst surface, which affects the severity of photodegradation.^107^^,^^118^ Several factors are known to influence the efficiency of photocatalysis, including catalyst properties, reactor design, light intensity, and contact time between the catalyst and pollutants.^107^ The band gap of a photocatalyst also plays an important role in its light absorption and photocatalytic reactions. For example, reduced graphene oxide (rGO-Gd_2_MoO_6_) contains a narrow band gap that improves its optical absorption capacity, and it is used in the photocatalytic degradation of pollutants in textile wastewater. The process is technically pseudo-first order reaction, in which the Langmuir-Hinshelwood model provides a way of expressing the rate of reaction in terms of the concentration of pollutants.^122^

ZnO is a photocatalyst that accelerates chemical reactions with the aid of light.^21^^,^^38^ ZnO, with a large band-gap energy, generates electron-hole pairs when it is exposed to sunlight, hence serving as an effective photocatalyst.^5^ Other than photocatalytic action, ZnO has applications in solar energy conversion, environmental cleanup, water and air purification, and sanitation.^99^^,^^123^ The process of photocatalysis of ZnO is such that on exposure to light, electron-hole pairs are created that cause redox and surface reactions.^124^ Electrons and holes that are photogenerated participate in oxidation and reduction reactions, which produce reactive species, i.e., hydroxyl and superoxide radicals that degrade pollutants.^82^^,^^125^

(Equation 1) ZnO + hv → e^−^ + h^+^

(Equation 2) O_2_ + e^−^ → O^−^_2_

(Equation 3) H_2_O + h^+^ → OH^−^ + H^+^

TiO_2_ is a semiconductor, and if it absorbs the light of width greater than or equal to the TiO_2_ band gap width, it leads to the formation of electrons and holes (e^−^– h^+^).^119^^,^^126^ The electron-hole separation is continued until the electron-hole pair travels to the surface of catalyst in which they participate in redox reactions.^127^ On this account, the interaction of h^+^_vb_ with water (surface-bound) forms the hydroxyl radicals, and oxygen selected by e^−^_cb_ yields the radical anion (superoxide radicals), as indicated below in Equations 4, 5, and 6.^116^

(Equation 4) TiO_2_ + hν → e^−^_cb_ + h^+^_vb_

(Equation 5) O_2_ + e^−^_cb_ → O_2_^−^

(Equation 6) H_2_O + h^+^_vb_ → OH + H^+^

**Figure 9:**
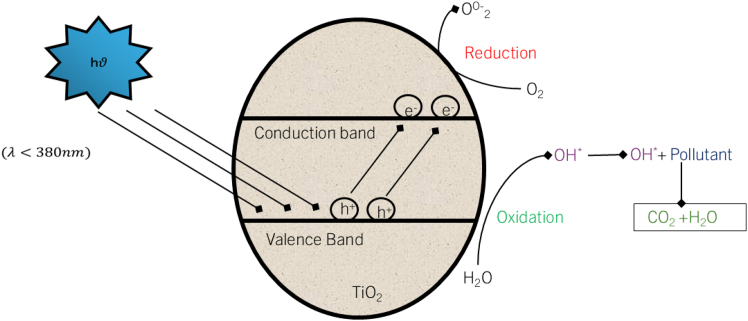
Process of the photocatalytic mechanism Reproduced with permission from Ghaly et al.^128^ Copyright 2011 Elsevier.

The main features of the photocatalytic mechanism (Figure 9) are based on the formation of electron-hole pairs and reactive species and the consideration of various factors involved in the degradation of pollutants.^122^

## Photocatalytic performance of nanomaterials

TiO_2_ and ZnO nanoparticles have excellent photocatalytic properties that breakdown the organic content of polluted water.^83^^,^^114^ Of these, TiO_2_ has been documented as having superior photocatalytic properties, stability, and ability to generate high concentrations of ROS in the UV-vis light, effectively mineralizing an extensive variety of pollutants, including dyes and heavy metals.^129^ However, the large band gap of TiO_2_ (3.2 eV) makes it difficult to absorb UV light, which is exciting for the electrode, while its response to visible light is lower.^26^^,^^100^ To overcome this, approaches such as metal doping have been undertaken to improve its photocatalytic properties under the visible-light regime.^5^ In contrast, ZnO (3.37 eV) offers the faster electron mobility and a higher quantum efficiency, though its tendency to undergo photo-corrosion restricts long-term reusability. Likewise, the photocatalytic properties for ZnO nanoparticles are enhanced, particularly when they are incorporated with polyvinylpyrrolidone. Recent investigations prove that light absorption of polymer and charge separation can be amplified by modifying the polymer surface (i.e., ZnO-PVP), which reduces recombination sites on the surface, resulting in better removal of organic pollutants like dyes and phenols. Different studies have shown that ZnO-PVP improves the reduction of COD and phosphate from municipal wastewater to levels of approximately 95% and 97%, respectively, with optimal UV light intensities and nanoparticle addition. Several studies have shown that UV-C light provides higher efficiency than UV-A or UV-B light in boosting photocatalytic processes.^114^^,^^130^ The incorporation of nanoparticles in wastewater treatment not only improves the rate of degradation of pollutants but is also a cheap and mobile technique for both on-site and off-site treatments of pollutants.^131^ The results for the degradation of different pollutants using different nanocatalysts are summarized in Table 2. New investigations are still directed toward the synthesis of nanocomposites and the integration of photocatalytic processes and membrane systems to improve the general effectiveness and efficiency of nanoparticle-mediated wastewater treatment processes.^7^

**Table 2:** Degradation of various pollutants by different nanocatalysts

Nanomaterial used	Pollutant type	Light source	Irradiation time	pH	Catalyst loading rate	Degradation efficiency	Reference
Biosynthesized ZnO NP	phenol	1,500 W xenon lamp	180 min	8	0.25 g/L	51%	El Golli et al.^109^
	*o*-cresol			7		52%	
	toluene			7		88%	
	xylene			7		93%	
Zn_0__.__5_Cu_0__.__5_Fe_2_O_4_	methylene blue	UV-C lamp	135 min	9	15 mg/L	94%	Abuzeyad et al.^117^
	methyl orange					96%	
ZnO-NPs	TDS	sunlight	7 h	8.7	1 g/L	76.4%	Haidri et al.^83^
	COD			7.7		57%	
	sulfates			7		81%	
	phosphates			7		67.3%	
MnTiO_2_-NPs	methylene blue	UV light source	100 min	9–12	0.1 g/L	78%	Awan et al.^49^
TiO_2_	methylene blue	UV light source	100 min	9–12	0.1 g/L	51%	Awan et al.^49^
nZVI	methylene blue	–	30 min	7–8	10 g/L	97.2%	Hamdy et al.^132^
rGO-ZnS-Ag nanocomposites	resorcinol	natural sunlight	150 min	7	0.5 mg/L	90%	Naeem et al.^101^
WO_3_ and TiO_2_	chemical oxygen demand (COD)	300 W xenon arc lamp	30 min	7	0.1 g/L	80%	Simelane & Dlamini,^104^
Agar-templated TiO_2_ coating	methylene blue	300 W xenon arc lamp	105 min	7	0.0011 g/L	62.4%	Najafidoust et al.^133^
ZnO:Ag nanoparticles	methylene blue	15 W UV lamps	90 min	–	30 mg/L	48%	Jadhav & Biswas^124^
	methyl orange	100 W tungsten lamp	120 min	–		86%	
Ni-doped ZnO nanostructure	methylene blue	UV lamp	30 min	–	50 mg/L	94%	Shkir et al.^99^
ZnO-PVP	phosphate (PO_3_-4)	UV-C lamp	7 h	7	1.5 g/L	97%	Abdolalian & Taghavijeloudar^114^
	(COD)					95%	
	crystal violet					95.38%	
ZnO nanoparticles (green tea-based, N-gZnO)	clomazone, tembotrione, ciprofloxacin, zearalenone	simulated solar	60 min	near neutral	0.5 mg/cm^3^	95.8–98.2%	Bognár et al.^134^
ZnO nanospheres (ZnO-S)	methylene blue	visible lamp (130 W)	90 min	neutral	0.05 g/L	98.64%	Porrawatkul et al.^135^
ZnO nanoparticles (calcined 400°C)	rhodamine B	UV, sunlight, visible	60 min	7	50 mg/L (S/L)	92% (UV), 91% (sunlight)	Saidani et al.^136^
ZnO thin film	methylene blue	UV lamp/UV laser	24 h (UV); 3 h (laser)	not specified	Thin film	74%	Atta et al.^137^
ZnO (*Justicia adhatoda*)	malachite green	UV & sunlight	180 min	not specified	0.64 g/L	99.8%	Mahajan et al.^138^
TiO_2_ nanoparticles	*p*-chlorophenol	UV	90 min	acidic	2.0 g/L	50.23	Hossain et al.^139^
TiO_2_ nanoparticles	tetracycline	UV lamp	Not specified	11 (best)	∼323 mg (reactor mass)	78.94% (optimized)	Zeinali Heris et al.,^140^
ZnO-TiO_2_ nanocomposite	malachite green	sunlight	sunlight (≤ full decolorization period)	5.8 (best)	not specified	92%	Nayak et al.^141^
ZnO-TiO_2_ nanocomposite	organic dye (methylene blue)	visible light	not specified	4	0.80 g/L	96%	Akhter et al.^142^
TiO_2_-ZnO nanocomposite (TZ-NC)	mixture of 6 antibiotics	UV & visible	<4 h	not specified	low dose	99%	Ansari et al.^143^
ZnO-TiO_2_ composite	methyl orange	low-power UV-A	not reported	6.7	0.2 g/80 mL	98.6%	Dai et al.^144^

The comparative results, as shown in Table 2, reveal a large difference in the photocatalytic degradation efficiency (48%–99.8%) with changes in the catalyst composition, type of pollutant, source of light, pH, and catalyst loading. ZnO-based materials, especially green synthesized or doped, are typically better performing. Indicatively, *Justicia adhatoda*-derived ZnO led to 99.8% degradation of malachite green under UV/sunlight, and N-doped green ZnO had 95.8%–98.2% degradation rate under simulated solar light.^134^^,^^138^ This increased efficiency can be explained by an increased surface area, reduced defect density, and decreased electron-hole recombination, which increase the generation of ROS. Composite formation and doping have a major effect on degradation kinetics. Ni-doped ZnO (94%) and ZnO-TiO_2_ nanocomposites (up to 99%) are better than pure TiO_2_ (≈50) as the formation of heterojunction allows charge separation and light absorption over a wider range including the visible region.^99^^,^^139^^,^^143^ Likewise, rGO-ZnS-Ag composites (90%) have an advantage of improved electron transport with the use of graphene and plasmonic effects of Ag nanoparticles.^101^

Variability is also explained by light intensity and light wavelength. UV-C sources and xenon arc lamps have higher efficiencies (e.g., 94%–96% Zn_0.5_Cu_0.5_Fe_2_O_4_) than the low-power UV lamps (48%).^117^^,^^124^ Under solar irradiation, band gap engineering increases the efficiency of visible light-active catalysts with respect to pH because changes in the catalyst surface charge and ionization of pollutants can be beneficial; in the case of cationic dyes, such as methylene blue, alkaline conditions (pH 9–12) are optimal. Some of these cases (e.g., thin films, 74% are lower in efficiencies due to low surface contact area as opposed to nanopowders.^137^ Catalyst loading also shows an optimum range; excessive loading may cause light scattering and reduced photonpenetration. Overall, the degradation efficiency is controlled by synergies between the catalyst architecture, band gap engineering, working conditions, and chemistry of the pollutant, with doped and composite systems based on ZnO having the most efficient and scalable photocatalytic degradation efficiency.

## Long-term environmental impacts or strategies to mitigate the toxicity of nanoparticles

ZnO and TiO_2_ nanoparticles pose long-term environmental and health hazards because they are persistent, highly reactive, and bioaccumulated. Chronic exposure has the ability to disrupt ecology and biological homeostasis, resulting in pulmonary, cardiovascular, and neurological disorders, immune malfunction, and inflammatory-related diseases.^84^^,^^121^ Nanoparticles can invade the walls of plants and prevent their growth, chlorophyll production, and photosynthesis rate, thus impacting crop productivity and the functioning of the ecosystem.^62^

In addition, TiO_2_ nanoparticles have demonstrated the ability to impede algal growth, suggesting potential ecological risks. However, for understanding the long-term ecotoxicological effect of the contaminants, the environmental interactions of the contaminants need further systematic studies.^33^ Like the last, ZnO nanoparticles raise serious environmental concerns like poor settling properties of activated sludge, and they could affect nitrogen and phosphorus removal efficiencies in wastewater treatment. ZnO nanoparticles are found to be reduced in toxicity by transformation, but their increased production brings more environmental concerns.^113^ ZnO nanoparticles and bioaccumulation and migration particles introduced in high concentrations in sewage treatment effluent have detrimental effects on the surface water, and the stability of these particles poses concern in their long-term efficacy. However, the toxicity of ZnO nanoparticles in drinking water treatment continues to be a serious problem and needs to be assessed as quickly as possible.^26^

In order to reduce these long-term risks, a number of measures have been suggested, including green synthesis approaches as a major preventive measure. Through the production of green nanoparticles using plant extracts and renewable biological materials, green synthesis removes solvents that are toxic and processes that require a lot of energy and produces biocompatible nanoparticles with enhanced stability and low cytotoxicity.^65^ Plant extracts can prevent aggregation and decrease ROS generation, as well as increase long-term colloidal stability by surface-bound biomolecules. Equally, silver nanoparticles that are transformed into sulfides have a much lower toxicity because increased solubility of silver sulfide reduces their environmental impact, demonstrating how transformation processes can be designed to reduce environmental risks.^63^^,^^71^ Other mitigation strategies are provided by surface engineering and functionalization. Controlling the size of particles, coating them with biodegradable polymers, or doping with elements like Fe may slow down the rate of dissolution, reduce the release of ions, and decrease ecotoxicity.^62^ Nanoparticles immobilized on solid surfaces facilitate recovery and reuse so that they are not released freely in the water system and are more sustainable in photocatalytic processes.^140^

An emerging method of sustainable bioremediation is the use of algal nanoparticles that do not produce toxic by-products during synthesis. They offer heavy metal and dye remediation, as well as environmentally friendly wastewater treatment.^17^ Modifications like encapsulating nanoparticles onto supporting materials can also reduce toxicities and enhance reactivity, and metal oxide doping mitigates the toxicities issues.^38^ Studies are ongoing to understand the transport and degradation mechanisms of nanomaterials; lifetime and toxic interpretation are possible only with stable morphologies.^116^

Last but not the least, toxicity studies in the long run in circumstances close to nature are much needed. In order to make nanomaterials sustainable, technological innovations should be combined with comprehensive ecotoxicology studies, regulatory policies, and effective disposal approaches.^61^ Green synthesis, surface modification, controlled deployment, and life cycle risk assessment present a middle way of enjoying the benefits of nanoparticles and reducing adverse effects on the environment and human health in the long term.

## Recent advancement of photocatalysis-based wastewater treatment

The advancement of photocatalytic nanoparticles in wastewater treatment has mainly aimed at improving nanoparticle performance and functionality.^11^ Nanoparticles have been investigated because of their large surface area, porosity, and catalytic ability to breakdown organic pollutants.^34^ These include photocatalytic semiconductors such as TiO_2_, Fe_2_O_3_, SiO_2_, ZnS, and ZnO, which are particularly effective photocatalysts for breaking down organic constituents under ambient conditions.^115^

In an optimum scenario of UV light, ZnO-PVP nanoparticles extracted through municipal wastewater showed a significant increase in photocatalytic destruction of COD and phosphate by 95% and 97%, respectively. Comparing the results with those reported for individual ZnO particles, it suggests that nanoparticle modification leads to superior performances in wastewater treatment.^145^ An economical and versatile alternative to traditional treatment methods is nanotechnology for both *in situ* and *ex situ* processes.^11^ It is well noted from previous studies that there is a strong correlation between removal efficiency, UV light intensity, and nanoparticle dosage. Such adjustments are possible at a low cost, thereby maximizing the treatment performance. Further investigations into active species formation during COD reduction and phosphate elimination further contribute to understanding the photocatalytic process with ZnO-PVP nanoparticles.^114^ Green synthesis of nanoparticles using plant extracts as reductants is also a new avenue for environmentally friendly sustainable wastewater treatment technologies.^34^

Magnetic-based composites (MBCs) and MNPs are increasingly being used in water remediation. These properties make them valuable for water treatment applications.^126^ This is because of their strong magnetic response, high contaminant removal rates, stability, and ease of reuse. However, surface modifications in MNPs further enhance the ejection of pollutants and the recyclability of MNPs.^146^ The application of nanotechnology in the treatment of wastewater is expected in future applications, such as disinfection processes, desalination, sensing, photocatalysis, and adsorption. For instance, TiO_2_ nanoparticles increase the surface wettability and antifouling properties of membranes, thereby improving the efficiency of water treatment. Advanced nanomaterials enable multifunctional performance in contaminant capture and elimination in nanocomposite membranes, thus improving process productivity.^26^

Recent technological achievements in nanotechnology have played a significant role in developing high-efficiency nanocatalysts stabilized by a vast variety of organic and inorganic ligands. These stabilizers are important in the amplification of catalytic activity, stability, and prevention of aggregation of nanoparticles. Organoselenium compounds have particularly been found to be extraordinarily effective because of their good coordination factor, redox peculiarities, and capacity to regulate the development of nanoparticles due to the capacity to form well-dispersed metal nanoparticles with high surface reactivity and stability.^147^^,^^148^

Compounds are flexible ligands that have a high affinity to metal surfaces in the distribution of particles and the availability of a greater number of active catalytic sites. Their redox activity also makes them efficient in electron transfer, and such nanocatalysts are very efficient in comparison with traditional systems.^148^^,^^149^ Another important use of these nanocatalysts is the reduction of nitroarenes to aminoarenes, which is a significant transformation in organic chemistry and industrial chemistry. Aminoarenes are important intermediates in pharmaceuticals, dyes, agrochemicals, and polymers. Organoselenium ligands stabilized metal nanoparticles like Au, Ag, Pd, Pt, and Cu have a high catalytic performance because of better dispersion and increased reaction with reactants.^150^^,^^151^

The typical reduction process uses sodium borohydride (NaBH_4_) where the nitro compound is reduced by the reducing agent through the nanocatalysts. The organoselenium stabilizers promote this reaction by facilitating the reaction by rendering the interface favorable to the reaction with mild conditions and increased sustainability.^149^^,^^151^ These nanocatalysts are also highly selective, recyclable, and deactivation resistant. The tight binding between selenium ligands and metal surfaces guarantees stability of the structure in the course of multiple cycles. Moreover, they are capable of working in mild conditions, which contributes to green chemistry by limiting the use of energy and the amount of waste produced.^147^^,^^148^

## Challenges and future directions

The problems associated with water pollution originate from organic pollutants, such as dyes from industries, especially the textile and dyeing industries, which affect the environment and are toxic and non-biodegradable.^152^ Challenges faced by the textile industry include an increase in toxic chemicals in the effluent and the need for immediate management of industrial effluent due to rapid industrialization. The color and organics in wastewater effluents from the textile industry cause heavy pollution. The integration of the right remedy measures involves the utilization of polymer-coated nanoparticles to overcome the challenges and minimize the pollution caused by industrial effluents.^11^ The aggregation of ZnO nanoparticles is due to their large surface area, which results in poor efficiency. The challenge in particle size reduction is to achieve a larger surface area and dispersion for better optical characteristics and catalytic applications.^125^ Wastewater treatment involves high maintenance costs, high production of biological sludge, operations by professional operators, and a long detention time.^114^ The difficulties associated with the periodic distribution of engineered nanoparticles (ENPs) in municipal WWTPs include gaps in the behavior of high-field nanoparticles and the length of time that the nanoparticles are transported.^24^ The main challenges are to understand the efficiency of ENP removal at various stages of water treatment processes and to estimate the strength of ENPs in municipal WWTPs.^153^ Precise control of the interfacial properties for optimizing the photocatalytic efficiency of ZnO:metal heterostructures remains a challenge, a deficiency in focused research efforts to determine the generative mechanism of reactive species under certain experimental conditions for ZnO:metal heterostructures. Organizing repeated, quantifiable, and affordable interfacial properties in ZnO:metal heterostructures remains a major challenge.^124^ Another problem that has been shown here is the problem of nanoparticle self-aggregation owing to van der Waals forces, which makes them less effective. Another challenge discussed is the cost effectiveness of the process of reusing nanoparticles after they have been used; this is a major concern when considering the practical use of nanoparticles in the treatment of water and wastewater. There is also insufficient understanding of the impact of nanoparticles on human health.^27^

Despite significant advances in nanotechnology-based wastewater treatment, several scientific, operational, and environmental challenges continue to limit the translation of laboratory-scale success to full-scale implementation. The most pressing issue is nanoparticle aggregation, which reduces surface area and light absorption efficiency, thereby diminishing the photocatalytic performance. Surface functionalization with polymers or surfactants and immobilization on magnetic or carbon supports have shown promise in maintaining dispersion and facilitating recovery, but long-term stability and cost effectiveness remain open questions.

Advances in technology with respect to nanomaterials support the prospects of photocatalytic wastewater treatment using nanoparticles. The degradation rate of pollutants and surface sensitivity improved owing to the large surface-to-volume ratio of nanoparticles. Optimization of these nanoparticles has been studied to increase hydrophilicity and reduce fouling, as is done with CNTs and TiO_2_ in membranes. The commercial application of these nanoparticles requires their synthesis according to the principles of green chemistry. Nanotechnology integration into hybrid membrane systems has great potential to overcome the limitations of pollutant removal, energy consumption, and space constraints. Furthermore, nanoparticles are tailored in such a way that they are suitable for applications such as recovering heavy metals and organic compounds. Green synthesis using microbes, fungi, and algae is a promising method to produce MN. Modifications in nanoparticle size and shape are better explored to optimize their properties and find greater applications in WWTPs. Evaluation of metal oxide nanoparticle (NP) safety for these microorganisms is necessary to delineate their potential impact on living organisms and the environment. Future research also aims to increase purification efficiency by improving existing wastewater treatment technologies using metal oxide nanoparticles. Energy intensity and fouling issues are being investigated using advanced generation membranes such as nanofibers and functionalized membranes. Scientific interest has grown in bio-enabled synthesis to develop economical and environmentally sustainable processes for creating nanoparticles. These include challenges such as low removal capacity and regeneration issues, which are being addressed by innovations such as microfluidic continuities and magnetic separators. The behavior and transport of these ENPs in ecosystems and WWTPs needs to be further analyzed in future research. Guiding implementation on a larger scale, evaluation of the efficiency of nanoparticle removal across the treatment phases, and the effects on the environment are performed. Finally, photocatalytic nanoparticles in wastewater treatment systems inevitably increase efficiency and reduce costs to cope with the global water pollution problems in the refinery, pharmaceutical, and vegetable oil industries.

From the research perspective, the engineering of band gap, building of heterojunctions (Z-scheme and S-scheme), and optimization of co-catalysts to make photocatalysts active in the visible spectrum should be the key directions in the future. In addition, agricultural or algal biomass green synthesis presents a pathway to sustainable large-scale production with low environmental impact. Lastly, the wastewater treatment schemes in the future ought to consider a circular-economy model wherein nanomaterials need to be recyclable and least toxic, and real-time ENP measurements of effluents should be implemented. Materials science and environmental engineering converge with the LCAs to ensure the conversion of nanophotocatalysts on paper into real-life implementation to the industrial level and economic viability and environmentally friendliness.

## Conclusion

Wastewater treatment procedures using nanometer-sized particles have great potential for pollutant decomposition via photocatalytic methods. ZnO and TiO_2_ are the most efficient due to their large surface areas that enable them to destroy organic compounds through the light drive. ZnO nanoparticles are superior to TiO_2_ photocatalysts because they can maintain a high rate of electron transport and carrier lifetime, leading to superior breakdown of pollutants in the case of UV light exposure. The addition of silver to ZnO nanoparticles forms Schottky barriers that enhance the electron-hole separation and formation of ROS as well as optimize the overall photocatalytic efficiency. The use of polymers on nanoparticles inhibits their aggregative behavior and preserves their activity levels, which are vital for wastewater treatment. The magnetic properties of Fe_2_O_3_ nanoparticles enable their use in pollutant adsorption and removal processes, although they present environmental and cost-related issues. The photocatalytic capabilities of the ZnO-PVP nanoparticles overshadow those of bare ZnO particles because the nanoparticles require a lower dosage when exposed to UV light for COD and phosphate reduction. Continuous monitoring of ENPs in WWTPs is essential because their concentrations change in the presence of nanoparticles. The large-scale application of these technologies encounters key barriers, including environmental effects, total costs, and dimensional limits. Polymers have been explored as coatings on nanoparticles for the treatment of waste from refineries, pharmaceutical producers, and the vegetable industry, and recyclability has been pursued. Nano-adsorbents made from iron demonstrate both magnetic characteristics and a high surface area, enabling economic water purification. The pollutant levels of TiO_2_ and ZnO vary seasonally because they reach their peak concentrations during the summer and winter months, respectively. The distribution of nanoparticles depends heavily on the organic and biological materials because primary and secondary sludge particles tend to trap a high percentage of ENPs in secondary sedimentation basins but exhibit seasonal variations in concentrations.

## Declaration of interests

The authors declare no competing interests.
